# Observations on Pulmonary Consumption, and on the Utility of the Climate of Madeira for Phthisical Patients, Addressed to a Physician in London

**Published:** 1801-04

**Authors:** J. Adams

**Affiliations:** Madeira


*Observations on Pulmonary Consumption, and on the Utility of the Climate of Madeira for Phthisical Patients, addressed to a Physician in London. By* Jos. Adams, M. D.

---

My dear Sir,

BEFORE my departure from England I had collected notes on many fubjects, which I conceived my leifure in this ifland would have enabled me to arrange. I need not tell you what I have compleated fince my arrival; if it feems little for five year's refidence, recollect that, healthy as this fpot is, it has furnifhed me with fome papers. At all events, you of all others fhould be the laft to accufe me of indolence, fince no man in the world is lefs difpofed than yourfelf to appear unprepared before the public. It is muoh to be wifhed that the author to whom you refer me, before he had made up his book on Confumption, had made clofer enquiries into what he only feems to hint, namely, the varieties of the difeafe. Perhaps, when this is accurately accomplifhed, inftead of wondering that Confumption is found in moft parts of the world, we fhall find even the climate of Great Britain a remedy for fome fpecies of that difeafe. Not, I will admit, where ulceration has taken place, becaufe a more equal temperature muft be neceffary, that the conftitution may be as little as poffible interrupted in repairing the mifchief. We have now too many proofs of the refources of Nature, to doubt her powers in healing ulcerated lungs. This, however, can only be under certain circumftances; for if fo important an organ has fuffered to fuch a degree as to prevent the neceſſary functions of life, the means of reſtoration are cut off, and the caſe muſt end fatally.

That what is called Phthiſis Pulmonalis is known all over the world cannot be doubted; but the true Engliſh Conſumption is, I believe, peculiar to cold, and chiefly to be dreaded in uncertain climates. It is worth while to mark the etymology of different countries. The Greeks gave the name φθισις, from the idea of corruption. Hippocrates, and his ſucceſſors, found in the lungs of ſome phthiſical ſubjects large collections of matter, which, as ſoon as the ſac had any communication with the air, became putrid. Hence they conſidered the diſeaſe a *corruption* of the lungs, and fancied that putrid matter from the liver and other parts, being transferred to that organ, might produce an incurable diſeaſe. We find Celſus, with his uſual accuracy, making a diſtinction between φθισις and *tabes*, conſidering the former as only one ſpecies of the latter.

But that ſpecies of conſumption from which originated the term phthiſis, is uſually the effect of pleuriſy, and is very different from another with which it is confounded, and which gave riſe to the idea that the expectoration of purulent matter was neceſſarily fatal. This laſt diſeaſe has its origin in the ramifications of the bronchia. It begins with cough and expectoration of mucus. If theſe continue for any time in a young ſubject, there is always an apprehenſion leſt the diſeaſe ſhould be confirmed; that is, leſt by frequent returns of inflammation the ſecretion ſhould become habitual. This danger is very much increaſed if the patient contracts the habit of ſtraining himſelf into a cough, in order to diſcharge a ſmall remaining quantity of mucus which he conceives will continue to irritate as long as it remains in the trachea, but which is in fact only ſecreted by the parts to protect them from the patient's efforts, conſequently in proportion to his diligence is the ſecretion increaſed. I have often been aſtoniſhed how little attention phyſicians have paid in not admoniſhing their patients to ſuppreſs their cough as much as poſſible. In all diſeaſed lungs this ſhould be attended to, but more particularly in the laſt mentioned; for by this conſtant irritation on a ſecreting ſurface, ulceration is at laſt produced, which, when we conſider the ramifications of the bronchia, may ſoon be ſo extenſive as to prove fatal. The only writer I find in my notes who deſcribes this ſpecies of conſumption is Chalmers, in his "Diſeaſes of Carolina;" it however exiſts, I believe, in moſt parts of the world, but principally where the ſeaſons are uncertain, and the inhabitants moſt ſubject to coughs.

A third cauſe of conſumption is not only found in every part of the world, but is much more common than is ſuſpected.

This

This is the only one that begins with that ſhort dry cough which many writers have conſidered as the firſt ſymptom of conſumptions in general. This diſeaſe is a chronic inflammation, or frequent habitual, though ſlight, inflammations of the lungs, which by repeated effuſion of coagulable lymph, produces adheſions of the cellular part of the lungs, and thus obliterates their cavity, or prevents their expanſion. The appearance in the dead ſubject is extremely well deſcribed by Dr. Baillie.* Mr. Abernethy, by his frequent examination of the bodies of thoſe who died phthiſical, detected it ſo often as to induce him to conſider it one of the moſt common cauſes of conſumptions.† This is, I believe, the only ſpecies of the diſeaſe known in this iſland, if we except thoſe from hæmorrhage and pleuriſy, both which are very uncommon.

Though all theſe are very diſtinct in their origin and progreſs, yet in the moſt advanced ſtages they have many ſymptoms in common; indeed, excepting the purulent expectoration, which never occurs in the conſolidated ſtate of the lungs from the adheſive inflammation, the cloſing ſymptoms of each are nearly ſimilar.

But you are growing impatient to hear of Madeira. True it is, my dear Sir, we are apt to be *ſemper ad eventum feſtinantes et in medias res*; and if I were writing only to you, the latter ought to be paſſed over *haud ſecus ac notas*; but you inſiſt on my writing to the world; if ſo, I muſt diſcriminate what I mean by a diſeaſe before I propoſe a remedy.

Mr. Abernethy, in the paſſage before alluded to, gives many judicious directions, by which the conſumption from conſolidated or infarcted lungs, if you will admit ſo antiquated an expreſſion, may be diſcovered at an early period. Whenever we find the ſhort dry cough with emaciation, it ſhould always be ſuſpected; and his teſt ſeems ſufficient to diſtinguiſh the diſeaſe from all others, excepting the early ſtage of numerous ſmall tubercles. To diſtinguiſh theſe two complaints we ſhould, in the latter, look for other ſigns of ſcrophula; but in the former, there is a peculiarity in the caſt and character of features which is very ſtriking. Inſtead of that ſenſibility which enlivens the ſcrophulous countenance, and that ſanguine diſpoſition which ſees, even in the moſt unfavourable ſymptoms, a proſpect of amendment, we find a ſtiffneſs in all the motions of the features, and of the whole body, which is always in a very erect poſture. The patient frequently anticipates his doom

* Morbid Anatomy, Chapter of the Lungs.

† Surgical and Phyſiological Eſſays, Part I. p. 155.

doom with a languor and complacency, if poffible, more affecting than the unfounded hopes of the other victim. When we are fatisfied that this is the difeafe, we may, I think, without change of climate, always infure fuccefs, at leaft as long as the appetite for food continues. Exercife, by which the blood is more determined to the limbs, and occafional evacuations to anticipate that plethora which may have become almoft periodically habitual, will feldom fail of fuccefs in any climate. But your patience muft be by this time exhaufted; I fhall, therefore, bring you to Madeira.

In all cafes of tubercular or fcrophulous confumption, if, as you exprefs it, the patient does not faunter away his time after you have advifed him to leave England, we can with certainty promife a cure.—Where the lungs are ulcerated from other caufes, it remains for you to determine whether there are powers remaining in the conftitution to effect a cure, if the patient is placed in the moft favourable circumftances; for though we fee many recover from a fituation which invariably proves fatal during the winter in England, yet we have alfo inftances in which an emaciated carcafe has been furrendered to the waves during the voyage, or arrived only early enough to be decently interred. In an earlier period of the difeafe there can be no fituation in the world fo well calculated for the reftoration of difeafed lungs as the ifland of Madeira.

The valley of Tunchall is defended by immenfe hills from every wind but the fouth, where it is open to the fea breeze; this preferves a temperature fo even, as is unknown in any other part of the world. Our winters may be compared to your fummers in every thing but the length of days, and thofe fudden changes from heat to cold to which you are fubject. The thermometer with us is often fteady within doors, or varies fcarcely a degree for weeks together. During winter, its whole range is from 58 to 65; and in fummer, from 70 to 75, rarely amounting to 80, the heat being always tempered by a breeze in proportion to the force of the fun. The drynefs of our atmofphere is not lefs remarkable; this is, I believe, of lefs confequence in confumptive cafes than in thofe which are called humoral afthma, a difeafe unknown in this country. For want of good hygrometers, we have hitherto only been able to judge by the abfence of fogs, by the rapidity of our rivers, which have refufed a nidus to all frefh water fifh excepting fuch eels as can fecure themfelves under large ftones, and by our fecurity from mufquitoes and moft other gnats; frogs, toads, and leeches are equally unknown. Since my arrival, I have not feen or heard of a cafe of intermittent fever; and the few dyfenteries produced by the autumn, are milder and more eafily relieved relieved than thofe in England. However, to decide the queftion beyond a doubt, I procured two of Mr. Lane's hygrometers: One of thefe was fufpended in open Veranda expofed to the beach, and the other at the refidence of the Hon. Auguftus Phipps, lefs than a mile out of town, and in a fituation generally reputed damp for this country. By Mr. Phipps's regifter, which you will receive with this, it appears that the finger rarely pointed higher than two, and was moft commonly lower for more than a month of our rainy feafon. The other hygrometer was fo perpetually at, or near °, that the gentleman who had the charge of it, grew tired of marking its trifling variations.

This difcuffion appears to me of no further confequence, than as far as truth is concerned, till it is found that a dry air is neceffary for thofe who feel a temporary relief from enhaling hydrogen gas, the fteam of water, and other analogous fubftances. The fact is much more to the purpofe, that in all cafes of fcrophulous confumption, not too far advanced, the climate of Madeira proves a certain remedy. The only obvious caufes I can offer for this *constant* fuccefs are, firft, the equal temperature of our climate; next, that the lungs are not irritated by any particles arifing from an open fire, or by the contraction of the fkin from a partial accefs of air, which artificial heat will always produce. Our roads too being moft of them paved, and no wheel carriages ufed in the moft inhabited part of the ifland, thofe clouds of duft never arife which dry weather produces in other parts of the world, which in hot climates will fometimes produce catarrh, and which are always found injurious to weak or difeafed lungs. Thefe are, I believe, the principal enquiries you wifhed to make: It is true, they are of little confequence compared to the important fact you have in view. It is, however, fatisfactory to trace probable caufes; and it may be well worth your while to try whether fpacious buildings, regularly heated, fafely ventilated, and large enough to admit of neceffary exercife, may not anfwer the purpofe for fuch whofe want of means, of courage, or of leifure, prevent their taking a voyage to a more genial climate. I remain,

My dear Sir,

Yours, faithfully,

